# DNA Translocation through Hydrophilic Nanopore in Hexagonal Boron Nitride

**DOI:** 10.1038/srep03287

**Published:** 2013-11-21

**Authors:** Zhi Zhou, Ying Hu, Hao Wang, Zhi Xu, Wenlong Wang, Xuedong Bai, Xinyan Shan, Xinghua Lu

**Affiliations:** 1 Beijing National Laboratory for Condensed-Matter Physics and Institute of Physics, Chinese Academy of Sciences, Beijing 100190, People's Republic of China

## Abstract

Ultra-thin solid-state nanopore with good wetting property is strongly desired to achieve high spatial resolution for DNA sequencing applications. Atomic thick hexagonal boron nitride (h-BN) layer provides a promising two-dimensional material for fabricating solid-state nanopores. Due to its good oxidation resistance, the hydrophilicity of h-BN nanopore device can be significantly improved by UV-Ozone treatment. The contact angle of a KCl-TE droplet on h-BN layer can be reduced from 57° to 26° after the treatment. Abundant DNA translocation events have been observed in such devices, and strong DNA-nanopore interaction has been revealed in pores smaller than 10 nm in diameter. The *1*/*f* noise level is closely related to the area of suspended h-BN layer, and it is significantly reduced in smaller supporting window. The demonstrated performance in h-BN nanopore paves the way towards base discrimination in a single DNA molecule.

As one of the most promising concepts, DNA sequencing with nanopore has attracted a lot of attention since 1996^1^. DNA translocation^1^,^2^,^3^ and base discrimination^4^ has been demonstrated in biological nanopores made of specific proteins. Solid-state nanopores, however, are strongly preferred for higher stability and easier device integration. The first solid-state nanopores are fabricated in silicon nitride (Si_3_N_4_) membranes that are tens of nanometers thick^5^, and significant progress has been made in detection of DNA translocation^6^,^7^, and DNA motion control^8^ in such nanopore devices. To achieve base discrimination in a single DNA molecule, the effective thickness of a solid-state nanopore has to be reduced down to sub-nanometer. For such propose, atomic thick graphene layers has been employed in fabricating nanopore devices featured with high resolution and geometrical sensitivity^9^,^10^,^11^,^12^. On the other hand, the hydrophilicity of nanopore is practically important to avoid clogging due to strong nonspecific hydrophobic interaction with DNA. Recently, the hydrophilicity of graphene nanopores has been improved by atomic-layer deposition of titanium dioxide^11^ and self-assembled hydrophilic coating^13^, which however increases the effective thickness of the pores. New types of atomic thick nanopores with high hydrophilicity are thus still strongly desired.

Hexagonal boron nitride (h-BN) layer, an atomic layered material similar to graphene^14^, is chemically stable with oxidation resistance^15^. In addition, it possesses merits of good thermal conductivity^16^, low dielectric loss and low conductivity (wide band gap energy of 5.97 eV)^17^. The single- and few-layer h-BN membranes have been obtained by cleavage^18^, epitaxial growth^19^,^20^, or chemical synthesis^21^, which provides a new material for nanopore applications. Recently, S. Liu *et al.* firstly reported the fabrication of solid-state nanopores based on few-layer BN membrane^22^, and totally about one hundred DNA translocation events have been observed. One of the reasons for the limited number of translocation events observed in BN nanopores is suggested to be the relatively low wetting ability in such material. Here, we show that the hydrophilicity of h-BN nanopores can actually be improved by UV-ozone (UVO) treatment and hundreds of DNA translocation events have been observed in single experimental runs. Our finding thus suggests further promise to utilize h-BN nanopore as ultra-thin solid-state device for future applications.

## Results

### Nanopore fabrication in h-BN layers

**Figure 1:**
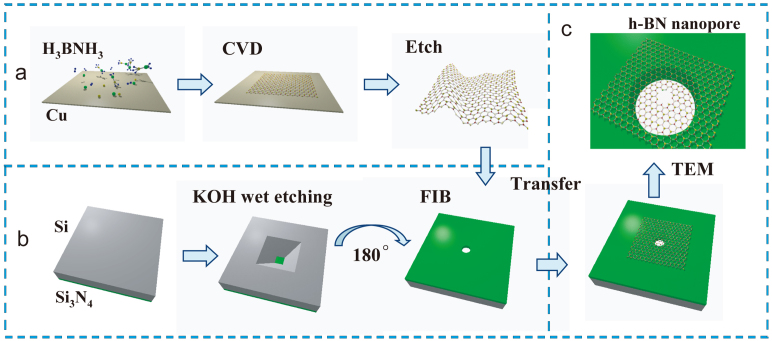
Fabrication process of h-BN nanopore. (a) h-BN membrane was first grown on copper foils by chemical vapor deposition. Then, PMMA was coated on h-BN membrane, and the copper substrate was subsequently etched by FeCl_3_, leaving h-BN/PMMA floating in solution. (b) Suspended Si_3_N_4_ window (shown as green) on a pyramidal silicon pit (shown as gray) was formed by anisotropic KOH etching. A 100–1000 nm sized hole was then drilled on Si_3_N_4_ window. (c) The floating h-BN/PMMA flake was transferred on to the window, and PMMA was removed by acetone. A nanopore was then fabricated on the suspended h-BN membrane in a TEM.

As sketched in Fig. 1, the single-layer h-BN membranes in our work were grown on copper foils (25 μm thickness, 99.99%, Alfa Aesar) by decomposition of borane ammonia (BH_3_-NH_3_, from Sigma Aldrich) under 1050°C^19^. Then, about 300 nm poly(methyl methacrylate) (PMMA) were coated on the h-BN membranes. After etching off the copper substrate in FeCl_3_ solution, floating h-BN/PMMA flacks (larger than 1 mm × 1 mm) were obtained. The floating flakes were carefully transferred onto a 50 nm thick Si_3_N_4_ window, which was prepared by KOH etching of Si_3_N_4_/Si wafer, as sketched in Fig. 1b. The size of the Si_3_N_4_ window is about 20–40 μm with a 100–1000 nm sized hole drilled by focused ion beam (FIB). Nanopore was then drilled on the suspended h-BN membrane with highly focused electron beam in a transmission electron microscope (Model: JEM 2010F).

### Characterization of h-BN nanopore

**Figure 2:**
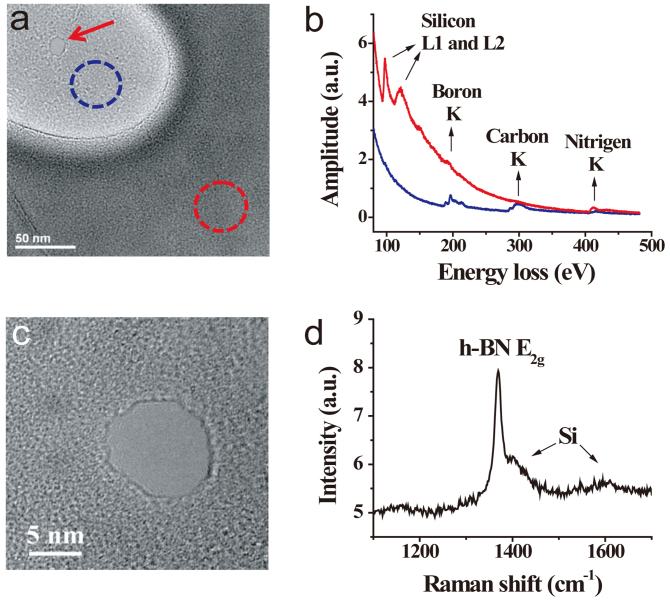
(a) TEM image of h-BN membrane (light gray area) on Si_3_N_4_ substrate (dark gray). The red arrow indicates an 8 nm nanopore. (b) EELS taken in regions of h-BN membrane (position indicated by blue circle in (a)) and Si_3_N_4_ substrate (red circle). (c) High resolution TEM image of a 10 nm h-BN nanopore. (d) Raman spectrum of h-BN layer transferred onto a Si substrate.

Figure 2(a) shows TEM image of a suspended h-BN layer (bright area) on Si_3_N_4_ window with an 8 nm nanopore, indicated with a red arrow. Electron energy loss spectra (EELS) were taken on both the suspended h-BN layer (position indicated with blue dashed circle) and the h-BN layer over the Si_3_N_4_ membrane (red dashed circle), as shown in Figure 2(b). Clear boron and nitrogen peaks appear in spectrum of suspended h-BN layer (in blue), while strong peaks of silicon and nitrogen are found in spectrum taken over the Si_3_N_4_ membrane (in red). The carbon peak in the spectrum of suspended h-BN layer is due to the residual amorphous carbon contaminated during layer transferring. Nanopores with diameter from 2 nm to tens of nanometers are fabricated (see supplementary information, Figure S1) in suspended h-BN layers. Figure 2(c) shows high-resolution TEM image of another nanopore (about 10 nm in diameter). The FFT of such TEM image shows clear six-fold symmetry (Figure S2), representing the hexagonal structure of the h-BN layer. Raman spectroscopy has been employed to further characterize the h-BN layers. Figure 2(d) shows typical Raman spectrum of h-BN layer transferred onto a silicon wafer (to minimize the background luminescence from Si_3_N_4_), excited with a laser wavelength of 532 nm. It is known that the E_2g_ peak of a single-layer h-BN is centered at 1369 ± 1 cm^−1^, while that for the bi-layer and the bulk are centered at 1365 ± 2 cm^−1^ and 1366 cm^−1^, respectively^23^. The observed Raman peak in our samples is centered at 1369.3 ± 0.1 cm^−1^ (Figure S3), indicating the presence of single-layer h-BN membrane. The small peaks at 1400 cm^−1^ and 1600 cm^−1^ are the third harmonic peaks of the silicon substrate^24^.

### Hydrophilicity improvement

**Figure 3:**
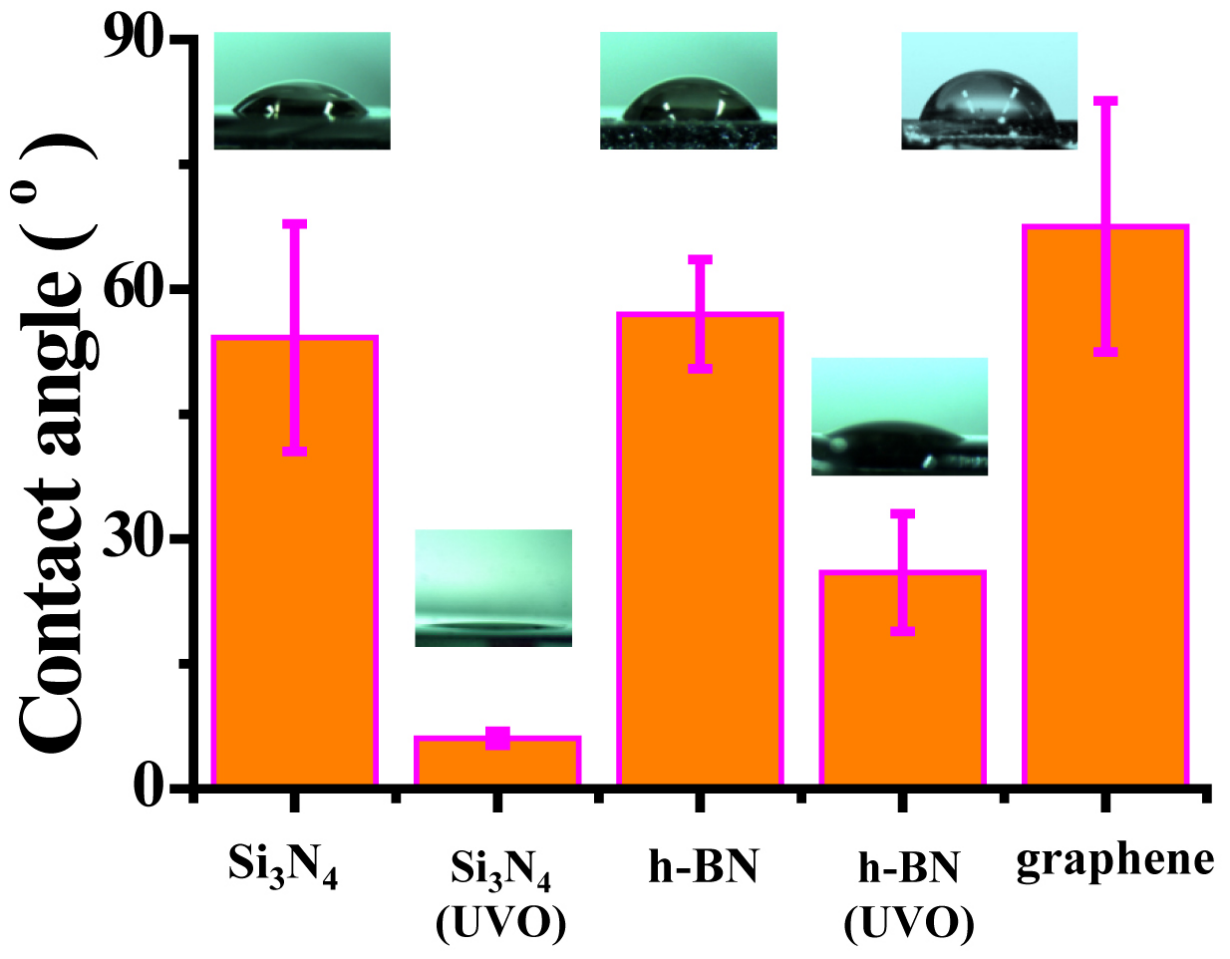
Contact angles of droplet (1 μl 1 M KCl-TE buffer) on surfaces of Si_3_N_4_ substrate (54°), UVO treated Si_3_N_4_ (6°), h-BN layer (57°), UVO treated h-BN layer (26°), and Graphene (67°). The insets are typical microscope images.

The h-BN material is resistant to oxidation and intact after UVO treatment, and such advantage can be used to improve hydrophilicity of h-BN nanopores. We characterized the wetting ability of h-BN layer by measuring the contact angle of a KCl-TE droplet on layer surface and compared it with other materials normally used for fabricating solid-state nanopores. The measurements are taken immediately after 15-minute UVO treatment (Jelight Company Inc., model No. 42-220). Figure 3 shows the measured contact angles on Si_3_N_4_ surface (50 nm thick), UVO treated Si_3_N_4_ surface, h-BN layer on Si_3_N_4_ substrate, UVO treated h-BN layer on Si_3_N_4_ substrate, and graphene on Si_3_N_4_ substrate. The UVO treatment was not applicable to graphene due to its low oxidation resistance^25^. Each measurement was carried out with more than 10 samples. The untreated h-BN layer gives a contact angle of 57°, close to that on Si_3_N_4_ surface (54°). The contact angle on UVO treated h-BN layer decreases to 26°. Such good wetting property can last for at least 30 minutes in ambient lab environment, long enough for device assembling in a typical nanopore experiment. We note that the UVO treated Si_3_N_4_ membrane shows the lowest contact angle of 6° and the graphene surface produce a contact angle of 67°, highest among all materials tested in our experiment.

### DNA translocation through h-BN nanopore

**Figure 4:**
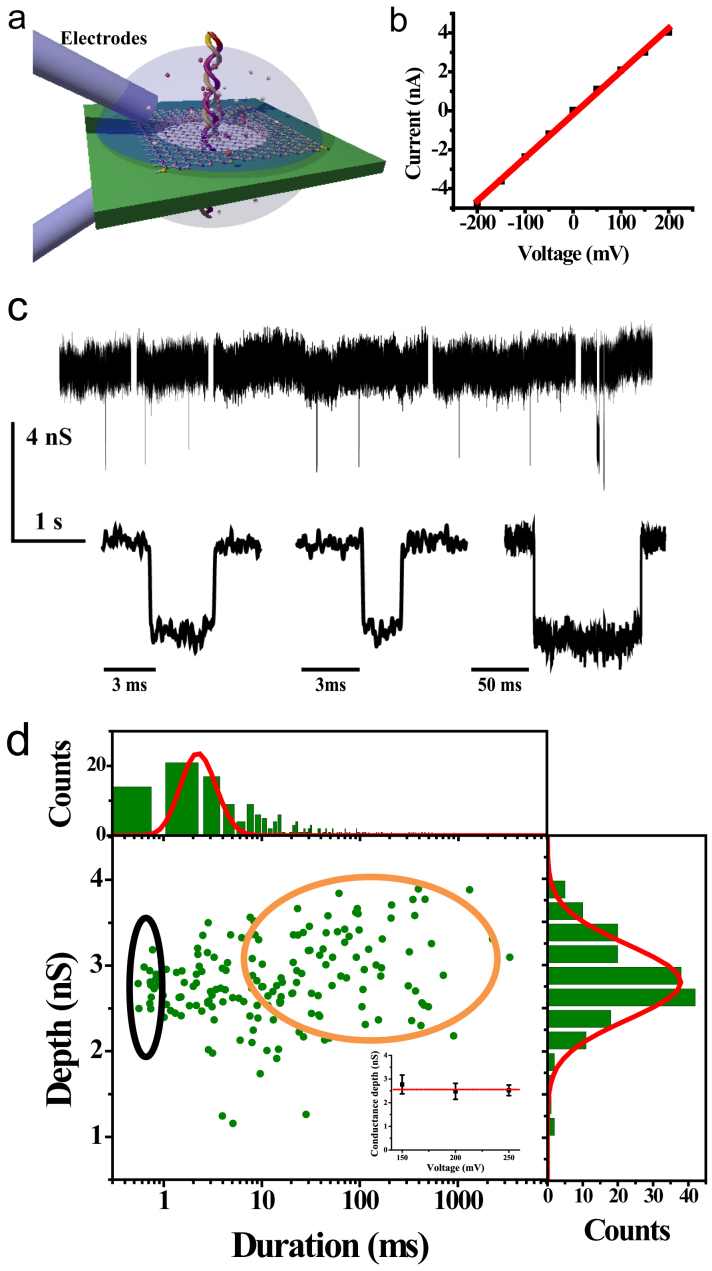
(a) Illustration of an h-BN nanopore device. (b) I-V curve measured in a 4 nm h-BN nanopore with a linear fit. (c) Current traces of DNA translocation in h-BN nanopore and zoom-in plots of typical translocation events. (d) Scatter plot of conductance blockade versus event duration, under a driving voltage of 150 mV. The histogram of conductance blockade is shown on the right side, fitted by a Gaussian peak. The histogram of event duration is shown on the top side, fitted by probability density function. The inset of (d) shows conductance blockade of unfolded events as a function of applied driving voltage.

To conduct the DNA translocation experiments, the flow cell, PDMS gaskets and the fabricated h-BN nanopore chips were firstly treated with UVO on both the front and the back side for 15 minutes each and then assembled as fast as possible (typically within 2 minutes). Saline solution (1 M KCl, room temperature, pH 7.8) was then added to both sides of the chip. A pair of Ag/AgCl electrodes supplied a bias voltage *V* to drive ions through the nanopore and the ionic current was monitored in real-time. Fig. 4(a) is a cartoon picture of such device, and Fig. 4(b) presents a typical I-V curve of a 4 nm nanopore in saline solution, through which a resistance of 45 MΩ is derived by a linear fit. When negative charged double-strand DNA molecules (λ-DNA from New England Biolabs) were added to one side of the chip, a series of spikes were observed in conductance traces (Fig. 4c), arisen from single DNA molecule translocation through the nanopore. The driving voltage is 100–250 mV and a 30 kHz 4-pole Bessel filter was employed during data acquisition and the sampling rate was 250 kHz.

Typically, at least hundreds of DNA translocation events can be observed in each nanopore device. Fig. 4(d) shows the scatter plot of conductance blockade ΔG versus time duration Δt of each DNA translocation event under driving voltage of 150 mV. The right panel is the histogram of conductance blockade, which can be fitted by a single Gaussian peak (blue line). The fitted curve is centered at 2.8 nS, corresponding to the averaged conductance blockade for unfolded DNA molecules. No translocation event of folded DNA was observed in this device since the pore size is too small for the folded DNA molecules to pass through. The averaged conductance blockade is invariant as the driving voltage changes from 150 mV to 250 mV, as shown in the inset of Fig. 4(d). On the upper side of the scatter plot is the histogram of translocation time Δt. To investigate the electrophoresis parameters of DNA translocation through the h-BN nanopore, we fit the events with density functions of the 1D based diffusion model^26^,^27^

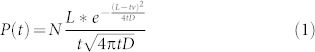
Where *N* is the normalized coefficient of the peak, *v* is the mean velocity of the molecules, and *D* is the diffusion constant. The passage length *L* = *L_DNA_* + *H_eff_* is simplified to the DNA length *L_DNA_* (16 μm for unfolded λ-DNA) because of the ultra-small thickness of the h-BN membrane. The fitting result is shown as solid red curve in the plot. The mean translocation speed for DNA translocation is 5.5 μm/ms, similar to that in previous report on λ-DNA translocation through graphene nanopore^10^. The diffusion constant *D* is fitted to be 8 μm^2^/ms, which is significantly larger than the bulk value of λ-DNA.

## Discussion

The histogram data do not match the fitting curve perfectly. Translocation events with duration time less than 1 ms (as contained in the black circle) may be contributed by DNA fragments, while those events with much longer duration time (contained in the orange circle) are due to DNA-nanopore interaction. For quantitative analysis, we discriminate elongated DNA translocation events as those events longer than the peak duration time by 1.5 σ, where σ is the full width at the half maximum (FWHM) of the fitted curve. The translocation time of elongated events in Fig. 4(d) is more than 6 ms. The percentage of elongated events adds up to 65% of all data points in the figure. The average translocation time of the elongated events is 108 ms, about 40 times longer than that of normal events. The anomalously long translocation time, as well as the high percentage, of elongated events indicates a pretty strong interaction between DNA molecules and the h-BN nanopore.

To further investigate such interaction in h-BN nanopores, we examined DNA translocation behavior in pores of different sizes. A moderate 10 nm (with λ-DNA) pore and a big 30 nm (with 10 kbp DNA) pore are fabricated for the test. Both folded and unfolded translocation events are observed in both pores (Figure S4). By analyzing duration time of all unfolded events (Figure S5), the percentage of translocation events undergoes molecule-pore interaction is about 71% in the 10 nm pore and about 20% in the 30 nm pore. The elongated translocation time is 16 ms (5 times of normal events) for the 10 nm pore and 0.85 ms (3 times of normal events) for the 30 nm pore.

**Figure 5:**
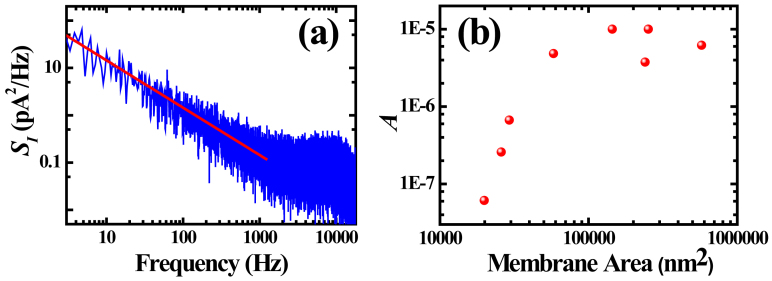
(a) Noise spectra of a 10 nm h-BN nanopore, taken from the current trace which contains DNA translocation events. The current baseline is around 15 nA, and the spectra is fitted by Hooge's law. (b) The value of fitted dimensionless parameter *A* as a function of suspended h-BN membrane area.

Figure 5(a) shows the noise spectrum of a typical h-BN nanopore on a 200 nm Si_3_N_4_ window, derived by FFT of real-time current traces. The low frequency noise can be fitted by Hooge's law: *S_I_* = A*I^2^/*f*, where *S_I_* is the power spectral density, *I* is the ionic current, *f* is the frequency, and *A* is a dimensionless parameter that can be used to characterize the 1/*f* noise level. The value of *A* is derived to be 6.7 × 10^−7^ for the 200 nm window. Interestingly, it is found that 1/*f* noise in h-BN nanopore is closely related to the size of the suspended membrane area. Figure 5(b) plots the value of *A* as a function of membrane area, which is obtained by subtracting nanopore area from the Si_3_N_4_ window area. For a smaller 180 nm window, the value reduces to 2.6 × 10^−7^; and for a bigger 550 nm window, it increases to 3.75 × 10^−6^. We noticed that the 1/*f* noise in a graphene nanopore is related to the size of the supporting Si_3_N_4_ windows as well^12^. Reducing the window size is not only good for optimizing the 1/*f* noise, but also help increase the yield of h-BN nanopores. In our practice, the h-BN nanopore device is very fragile if the supporting Si_3_N_4_ window is bigger than 500 nm, while most nanopores were stable when supported by Si_3_N_4_ window smaller than 200 nm.

In summary, we have demonstrated the nanopore device fabricated on atomic thick h-BN layer. The h-BN nanopores are of good oxidation resistance and are compatible with UVO treatment. The hydrophilicity of h-BN nanopores is improved by UVO treatment with a reduced contact angle of 26°. Abundant DNA translocation events have been readily detected in such nanopore devices with various pore sizes. Strong DNA-nanopore interaction has been revealed in small nanopores, which slows down the DNA translocation significantly. Smaller supporting windows are desired for h-BN nanopores to reduce the 1/*f* noise and to improve device stability. The demonstrated performance in such hydrophilic ultra-thin h-BN nanopores suggests further promise towards DNA base discrimination based on ionic current analysis.

## Methods

### Growth of h-BN

Hexagonal boron nitride was grown on a copper foil (25 μm thick, 99.99%, from Alfa Aesar). After cleaned by acetone, ethanol, isopropanol, and deionized water, the copper foil was placed into a quartz tube at 1050°C, where borane ammonia (BH3-NH3, from Sigma Aldrich) was carried by mixed gas of Ar and H_2_ (200/200 sccm) under atmospheric pressure. Hexagonal BN layer formed on the surface of copper foil after 30 min of growth.

### Transfer of h-BN

PMMA solution (PMMA 495, concentration 5%) was spin-coated on one side of h-BN/copper foils at 3000 rpm for 60 s and cured at 145°C for 5 minutes. The 3 × 3 mm^2^ Cu substrate was then etched away by iron chloride (1 mol/L) over a period of 12 hours, leave h-BN/PMMA flakes floating in solution. The flakes were then picked up, washed with deionized water several times, and then transferred onto the target substrates. After transfer, PMMA was dissolved with acetone and the substrate with h-BN layer was rinsed by isopropyl alcohol and dried with nitrogen gas flow.

### Nanopore fabrication and characterization

The nanopores were drilled by TEM (JEM 2010F, operated at 200 kV), focusing on h-BN membranes for about 1–3 s under about 500 pA/cm^2^ current density and a magnification of 800 k. After drilling, the electron beam was defocused to take images as quickly as possible. The EELS were taken subsequently with a magnification of 100 k and a diaphragm of 100 nm, under the energy resolution of 0.3 eV and integration time of 1.0 s. For each spectrum, the object astigmatism and zero-peak drifting were carefully calibrated. The pore size can be elaborately controlled. To measure the Raman spectra, the h-BN membrane was transferred onto a silicon wafer in order to avoid strong luminescence from Si_3_N_4_ substrate. All Raman spectra were taken under 532 nm laser excitation, with focal size of 2 μm and power of 1 mW (HORIBA, JY6400).

### Nanopore experiments

The flow cell, PDMS gaskets and nanopore chips were firstly treated with UVO on both the front and the back side for 15 minutes each and then assembled as soon as possible. KCl-TE buffer (contain 1 M KCl, 10 mM Tris-HCl, 1 mM EDTA, pH = 7.8) was filled into the device immediately. Subsequently, the BN nanopores were characterized by I-V curve, and only those chips providing linear and symmetry *I-V* curves were selected for DNA translocation experiment. Both double strand λ-DNA (48.5 kbp) and 10 kbp DNA were diluted to 1 nM by KCl-TE buffer and pre-warmed to 70° for 1 minute to activate DNA molecules. After injection of DNA buffer, driving voltages between 50 mV and 250 mV were applied across the membranes. The ionic current was detected by an Axopatch amplifier (200B) at acquisition rate of 250 kHz, with a 30 kHz 4-pole Bessel filter. The recorded translocation events were processed in a Matlab GUI program. The program allows us to view the events one by one and only those events with signal-noise-ratio (SNR) bigger than 8 and have a sharp edge are selected for analysis.

## Author Contributions

Z.Z. fabricated nanopores by FIB and TEM, and run the DNA translocation experiments. Y.H. transferred the h-BN layers and built the flow cell setup. H.W. and W.W. prepared the h-BN sample by CVD. Z.X. and X.B. performed TEM experiments and give helpful guide. Z.Z. and X.S. analyzed the data and X.L. designed and initiated the experiments. Z.Z., X.S. and X.L. were mainly responsible for the preparing of the manuscript with discussion and comments of other authors.

## Supplementary Material

Supplementary Information DNA Translocation through Hydrophilic Nanopore in Hexagonal Boron Nitride
