# Supplementary material for: DNA Translocation through Hydrophilic Nanopore in Hexagonal Boron Nitride
Source: Sci Rep. 2013 Nov 21;3:3287. doi: 10.1038/srep03287 (PMC3836030; doi:10.1038/srep03287)
Supplement: Supplementary Information — DNA Translocation through Hydrophilic Nanopore in Hexagonal Boron Nitride [file srep03287-s1.pdf]

Supplementary information

DNA Translocation through Hydrophilic Nanopore in Hexagonal Boron Nitride

Zhi Zhou, Ying Hu, Hao Wang, Zhi Xu, Wenlong Wang, Xuedong Bai, Xinyan Shan*, Xinghua Lu*

Beijing National Laboratory for Condensed-Matter Physics and Institute of Physics, Chinese Academy of Sciences, Beijing 100190, People's Republic of China

*Corresponding author: xhlu@iphy.ac.cn, shanxinyan@iphy.ac.cn

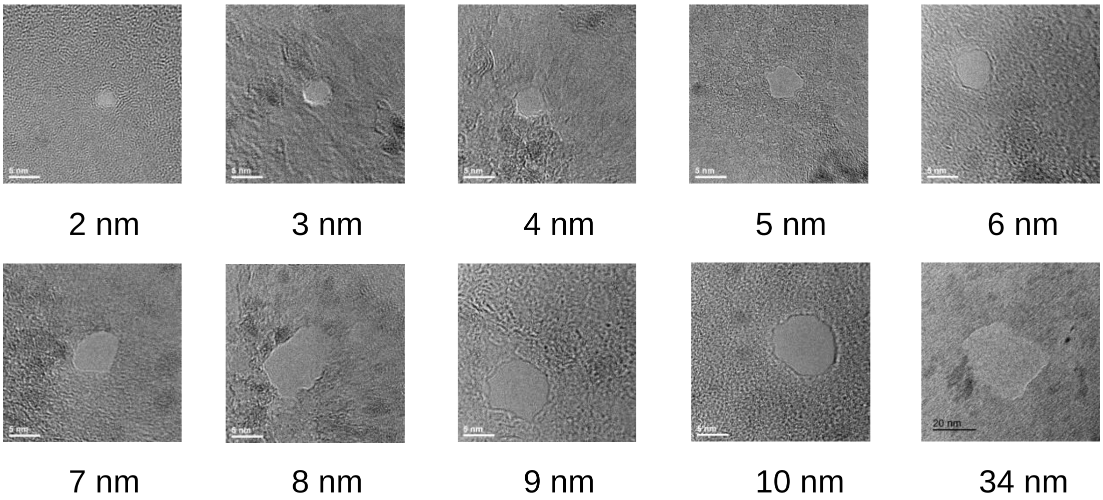

Figure S1. TEM images of typical h-BN nanopores with diameter from 2 to 34 nm.

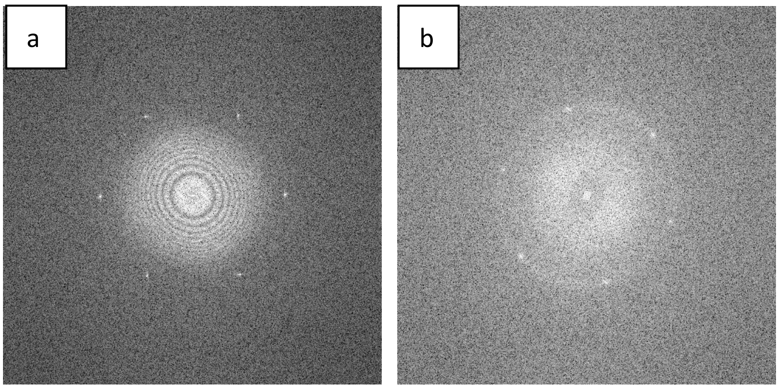

Figure S2. (a) FFT of Fig. 2(c) in the main text. (b) FFT of another full h-BN membrane's TEM image. Both show clear six-fold symmetry representing the hexagonal structure of the h-BN layer. The circular pattern in (a) is due to the over-defocus of the electron beam.

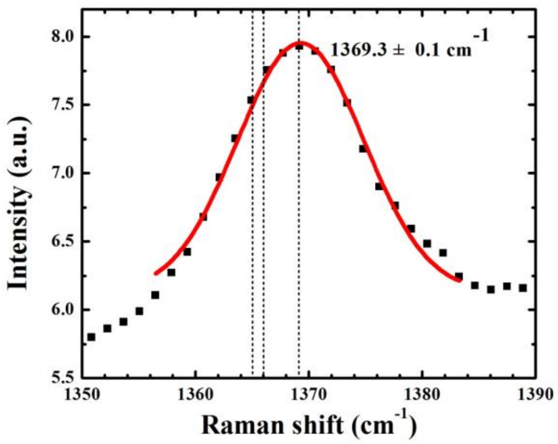

Figure S3. Zoom-in Raman spectrum of E_{2g} peak of h-BN layer. The peak centred at 1369.3 ± 0.1 cm^{-1} as derived from fitting with a Gaussian line profile. The dashed lines for eye guide at 1365, 1366, and 1369 cm^{-1} indicate the peak positions for bulk, double layer, and single layer h-BN, respectively.

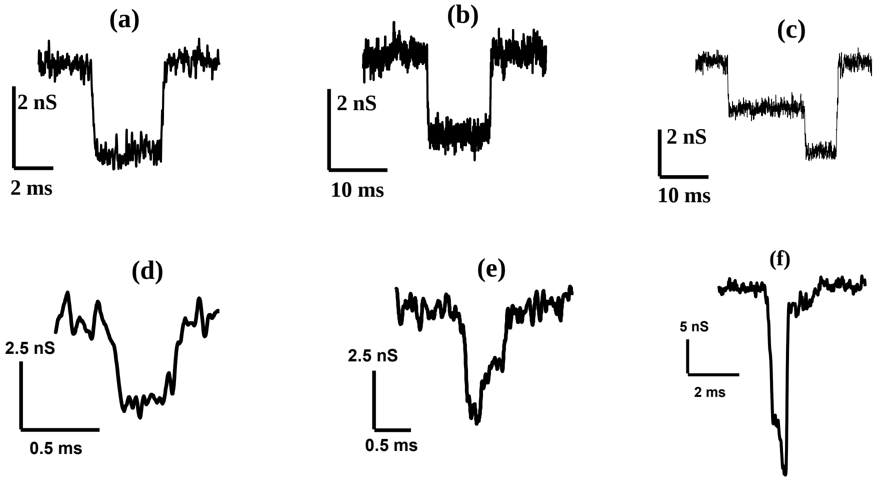

Figure S4. Typical translocation events through a 10 nm h-BN nanopore (a-c) and a 30 nm h-BN nanopore (d-f). Both unfolded (a, b) and partial folded (c) events are observed in the 10 nm pore. Unfolded (d), partial folded (e), and even multiple folded (f) events are observed in the 30 nm pore.

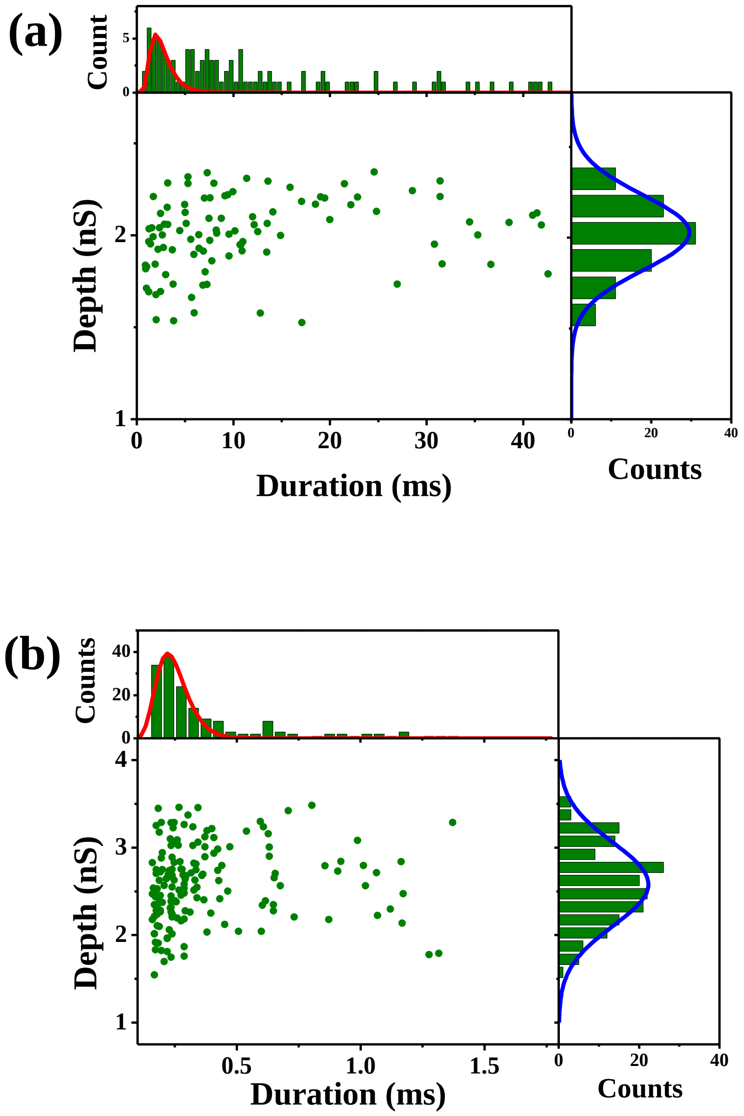

Figure S5. Scatter plot of conductance blockade versus duration time for unfolded events in a 10 nm (a) and a 30 nm (b) h-BN nanopore. The folded and partial folded events are manually screened out from original data. The conductance histogram is plotted at the right side and fitted by a Gaussian peak (shown as blue lines). The duration histogram is plotted on the top and fitted by the 1D bias diffusion model (shown as red lines).
